# Climate Change and Mental Health in Africa: A Scoping Review

**DOI:** 10.5334/aogh.5110

**Published:** 2026-01-14

**Authors:** Beverly N. Ndifoin, Ulrick Sidney Kanmounye, Kennedy Kwami Edem Kukuia, Francky Teddy Endomba, Aimé Gilbert Mbonda Noula, Desmond T. Jumbam

**Affiliations:** 1 Eco Game Changers, Yaounde, Cameroon; 2 Global Health Unfiltered, Accra, Ghana; 3 University of Ghana Medical School, Accra, Ghana; 4 Department of Medical Pharmacology, University of Ghana Medical School, University of Ghana, Accra, Ghana; 5 Université Bourgogne Europe, CHU Dijon Bourgogne, Service de Psychiatrie Adultes, 21000 Dijon, France; 6 INSERM, Center for Translation and Molecular Medicine (CTM), Team PAthophysiology of DYSlipidemia (PADYS), Dijon, France; 7 University of Yaounde I, Public Health Department, Yaounde, Cameroon

**Keywords:** climate change, mental health, global health, sub-Saharan Africa

## Abstract

*Background:* Climate change-related events such as floods, droughts, and wildfires have been shown to affect global mental health. As climate change worsens, extreme weather events increase, leading to more climate-related mental health disorders globally.

*Objective:* This review article assesses the impact of mental health and climate change in Africa to identify trends, research gaps, and potential interventions.

*Methods:* A scoping review methodology, in accordance with the PRISMA-ScR guidelines, was employed. A search strategy was developed using MeSH and synonym terms to search PubMed, Web of Science, and African Journal Online databases from January 2000 to April 2025. A total of 2332 titles and abstracts were screened.

*Results:* Sixteen articles were included in our final analysis. The studies included were conducted in three East African countries, three North African countries, two West African countries, two Central African countries, and one Southern African country. They were published between 2015 and 2024. Most (56%; *n* = 9) of the studies were cross-sectional studies. Climate change-related events, such as flooding, drought, and sea-level rise, have been found to affect mental health outcomes in countries like Ghana, Namibia, Nigeria, and Kenya. Commonly cited mental health outcomes included higher anxiety levels and lower well-being among relocated individuals, persistent stress and anxiety due to flooding in Ghana, and significant post-traumatic stress disorder symptoms among schoolchildren in Namibia. Vulnerable populations like children, adolescents, women, climate migrants, people living with HIV, and rural populations were found to be most impacted by climate change-related events.

*Conclusion:* While this review highlights an increasing trend in the impact of climate change on the mental health of individuals in Africa, more studies are necessary to establish the relationship between mental health and climate change, and to develop interventions and policies that address the growing mental health burden resulting from climate change.

## Introduction

It has long been understood that human activities, such as burning fossil fuels like coal, oil, and gas, contribute to long-term changes in global temperatures and weather patterns, which in turn impact both planetary and human health [1, 2]. Consequently, the World Health Organization (WHO) has declared climate change one of the biggest threats to global health [3]. Similarly, the Lancet Countdown on Health and Climate Change declared climate change the greatest global health challenge facing the 21st century [4]. These conclusions are understandable, as research studies from around the world have established a link between climate change and communicable diseases, including vector-borne diseases such as malaria, dengue fever, Lyme disease, West Nile virus, and Chagas disease [5, 6]. Climate change has also been linked to non-communicable diseases, including cardiovascular diseases, respiratory disorders, and heat-related illnesses, which are projected to increase in frequency and severity as temperatures rise and air quality declines [2, 7–9]. In Africa, climate change is exacerbating these health challenges while simultaneously intensifying water stress, damaging agricultural production, widening social inequalities, and threatening the livelihoods and well-being of communities [10]. Recent data underscore the severity of this crisis, with 2024 being ranked as one of the warmest years on record, accompanied by record-high sea surface temperatures and extreme weather events, including droughts and floods [11].

Increasingly, research is focusing on the health impacts of climate change [12–14]. Although most research has focused on the effects of climate change on physical health outcomes, a rapidly growing body of research is now exploring the effects of climate change on mental health [15]. Such research is crucial because mental health disorders already account for a significant proportion of the global burden of disease, and climate change stressors are likely to exacerbate these outcomes [16].

**Figure 1:**
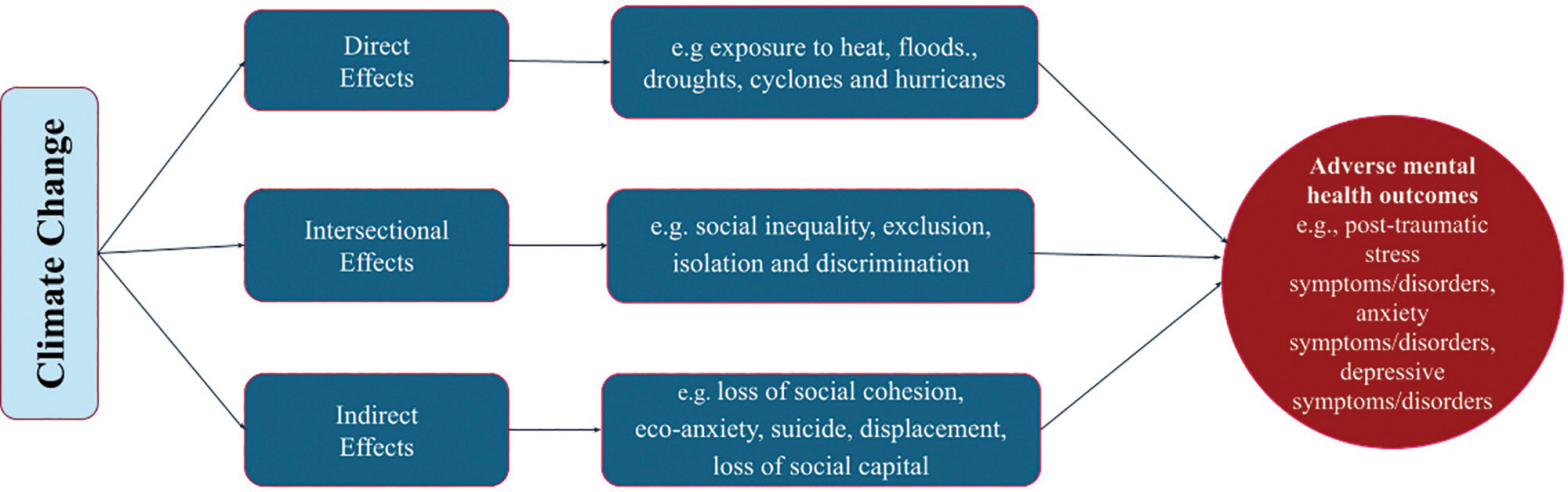
Direct, indirect, and intersectional ways in which climate change adversely impacts mental health outcomes. Framework adapted from Heinz & Brandt [18].

An overview of the current literature suggests that climate change is impacting global mental health in direct, indirect, and intersectional ways [7, 12, 17]. Direct effects of climate change on mental health include psychological trauma from experiencing extreme weather events like wildfires, floods, hurricanes, and droughts (Figure 1) [18]. Indirect pathways are through economic losses, displacement, food insecurity, disruption of social and community networks [18], eco-anxiety, distress, fear, and worry, especially among children and young people, as eco-anxiety is consistently associated with psychological distress, depression, and stress symptoms [19, 20]. Heinz and Bradt highlight an intersectional pathway where climate, health, and social inequalities converge [18]. At this intersection, vulnerable populations, including those with fewer socioeconomic resources, existing mental health disorders, and heightened exposure to climate-related environmental stressors, may be especially at risk for worsening health and social conditions [18, 21]. These stressors are closely tied to social, political, and economic determinants of health, such as poverty, unemployment, gender-based vulnerabilities, and inadequate access to healthcare [17]. For example, studies in Ghana and Nigeria have shown that exposure to floods is associated with higher rates of psychological distress, depression, and anxiety among both adults and children [22, 23]. Smallholder farmers in Kenya also reported experiencing emotional distress and hopelessness due to declining agricultural productivity resulting from droughts and unpredictable weather patterns [24].

As climate change worsens, the frequency and intensity of extreme weather events are expected to increase [25]. Consequently, climate-related mental health disorders like depression, post-traumatic stress disorder (PTSD), and climate anxiety are predicted to increase [26, 27]. Young people are particularly vulnerable to climate-related mental health disorders [20, 26, 28]. Emerging evidence also suggests an association between climate change and suicidality, with high temperatures and air pollution linked to high suicide attempts, especially among males [29]. This link could be explained by physiological factors like heat stress affecting brain function, psychological factors worsening depression and anxiety, and emotional responses such as eco-anxiety and hopelessness about the future of our planet [30].

Research has also shown that vulnerable people in low- and middle-income countries (LMICs) bear the brunt of climate change impacts. The WHO estimates that 99% of the disease burden of climate change is occurring in LMICs, and 88% of that burden affects children under the age of five [31]. In sub-Saharan Africa, where health systems are already strained, the growing threat of climate-related psychological stressors could be a major challenge for already strained mental health systems and policymakers.

The purpose of this review was to assess the existing literature on mental health and climate change in Africa, identify key trends, and pinpoint research gaps. By synthesizing studies from diverse African contexts, this review seeks to inform future research agendas, strengthen advocacy for mental health services, and support the development of integrated, climate-resilient health systems. Ultimately, the findings from this scoping review will support the design of more targeted, inclusive, and equitable climate-health adaptation strategies and policies across the continent.

## Methods

### Study design

This review was conducted using the scoping review methodological framework proposed by Arksey and O’Malley and later refined by Levac et al. [32, 33]. The review follows the Preferred Reporting Items for Systematic Reviews and Meta-Analyses for Scoping Reviews (PRISMA-ScR) guidelines. BNN and DTJ created search strings (facilitated by Claude.ai from Anthropic) to search PubMed, Web of Science, and the African Journal Online to identify original studies on climate change and mental health in Africa. Claude.ai assisted in generating search strings only, without influencing data interpretation. The string search terms were tailored for each database. The title, abstract, and full-text screening were conducted independently by BNN and DTJ using the Rayyan systematic review tool to reduce individual screener bias, minimize errors of omission, and increase reliability of the screening process. Each reviewer followed the inclusion and exclusion criteria to determine which studies to include in the analysis. Heinz and Brandt’s framework on the effects of climate change on mental health was used to categorize the study’s results [18].

### Research questions

The review was guided by the following questions: (1) What is the current state of research on climate change and mental health in Africa? (2) What gaps exist in the current research on climate change and mental health in Africa?

### Inclusion criteria

Studies were included if they met the following criteria:

- Peer-reviewed primary research articles
- Focus on one or more African countries
- Explicit examination of the relationship between climate change (or climate-related variables/events) and mental health outcomes
- Published between 2000 and the present
- Studies with quantifiable mental health variables or qualitative assessments
- Studies published in the English language

### Search strategy

To identify relevant articles on climate change and mental health in Africa, we conducted a comprehensive search spanning database inception to April 2025 using two main electronic databases: PubMed/MEDLINE and Web of Science. Our search strategy was built around three key areas: (1) climate change phenomena, (2) mental health outcomes, and (3) the African geographic context. We used different search strings with keywords customized to the database, as shown in Supplemental File 1.

This scoping review was reported per the PRISMA-ScR (Supplemental File 2) [34].

## Results

**Figure 2:**
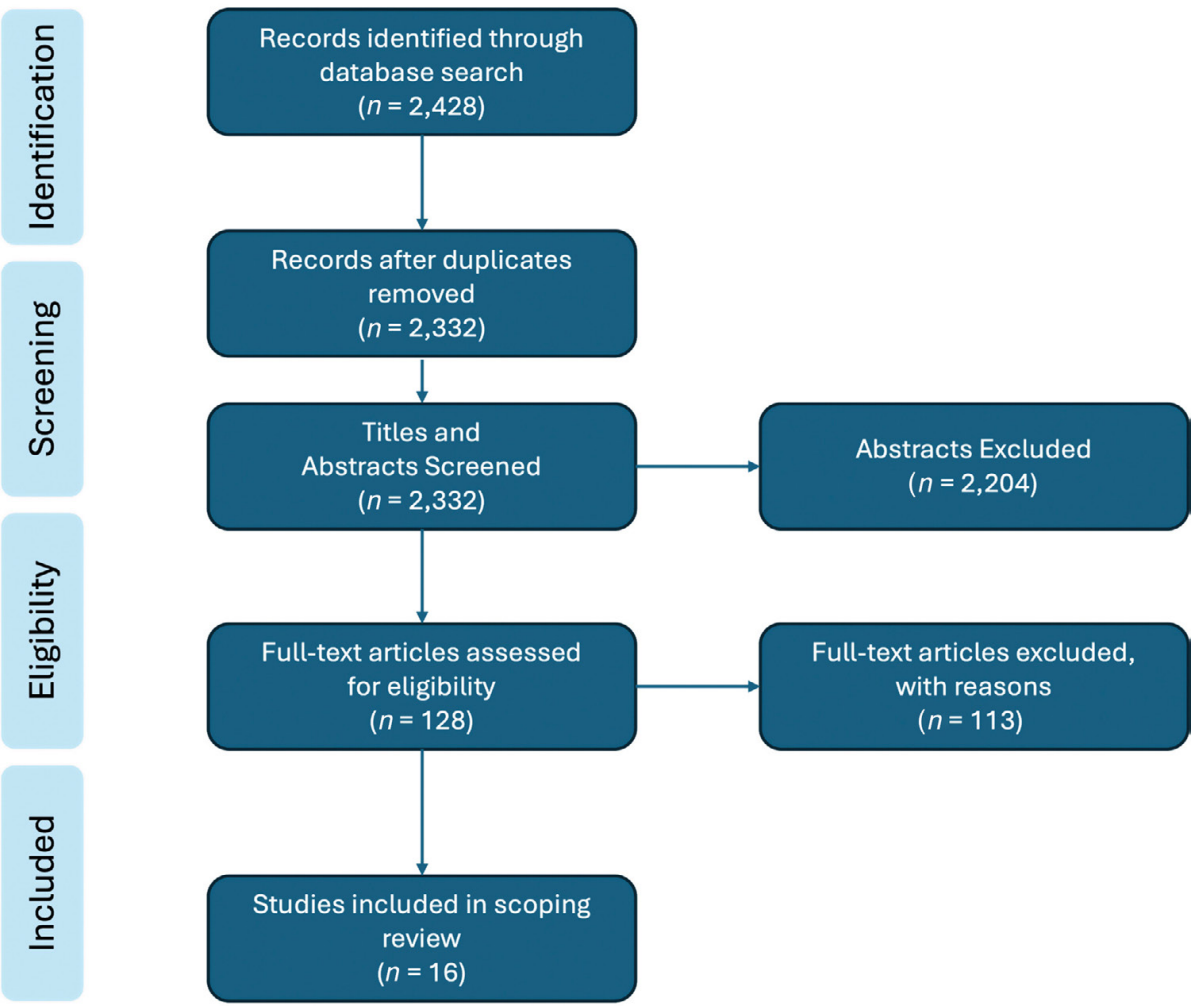
PRISMA flowchart of study selection.

We found 128 potential studies after screening 2332 titles and abstracts during the first round of screening. A total of 16 articles were included in our final analysis after full-text review in the second round of screening. Figure 2 presents the study selection process using a PRISMA flowchart.

### Overview of included studies

The studies included in the review were conducted in East Africa (Kenya, Tanzania, and Rwanda), West Africa (Ghana and Nigeria), Southern Africa (Namibia), North Africa (Egypt, Morocco, and Algeria), and Central Africa (the Democratic Republic of the Congo and Gabon). Most studies were published between 2019 and 2024. There was an increasing number of studies focused on climate change and mental health in Africa, with only four studies published between 2015 and 2020 compared to 12 studies between 2021 and 2024. Most (56%; *n* = 9) of the studies included were cross-sectional studies, two were randomized controlled trials, and four were qualitative and mixed methods studies. One policy commentary was included. The key results from the review are summarized in Table 1.

**Table 1:** Summary of Studies Included in the Review.

AUTHORS	REFERENCES	LOCATION	POPULATION	CLIMATE EVENT	MENTAL HEALTH OUTCOME	STUDY DESIGN	INTERVENTION
Heaney & Winter	[35]	Tanzania	Maasai migrants	Drought	Stress, loneliness	Qualitative	None
Taukeni et al.	[36]	Namibia	Children (8–18)	Floods	PTSD	Cross-sectional	None
Korukire et al.	[37]	Rwanda	General population	Floods, storms	Mental illness management model	Policy commentary	None
Di Giorgi et al.	[38]	Italy (African migrants)	Migrants	Chronic stressors	Climate perception & emotional disorders	Cross-sectional	None
Adams & Nyantakyi-Frimpong	[39]	Ghana	Flood-affected urban poor	Floods	Stress, anxiety	Photovoice	None
Hickman et al.	[20]	Nigeria (global study)	Youth (16–25)	Climate change	Climate anxiety	Cross-sectional	None
Abass et al.	[22]	Ghana	Adults in flood-prone areas	Floods	Psychological distress	Cross-sectional	None
Heeren et al.	[40]	Gabon, Rwanda, etc.	General adult population	None	Climate anxiety	Cross-sectional	None
Ugwuoke et al.	[41]	Nigeria	Farmers	Floods	Anxiety disorders	RCT	REFHT intervention
Beyeler et al.	[24]	Kenya	Farmers with HIV	Drought, Floods	Emotional distress	Qualitative	None
Obaniyi et al.	[42]	Nigeria	Rural farming households	Extreme weather	Stress, anxiety, depression	Cross-sectional	None
Ndetei et al.	[43]	Kenya	High school students	Floods, Droughts, Heat	Mental health & suicidality	Cross-sectional	None
Atta et al.	[44]	Egypt	Nursing staff	None	Climate anxiety & job engagement	Cross-sectional	None
Anichebe et al.	[45]	Nigeria	Children (10–16)	Floods	Anxiety & PTSD	RCT	Drama & Music Therapy
Balakrishnan et al.	[46]	Kenya	Women in informal settlements	Extreme weather	Stress & anxiety	Mixed methods	None
Abu et al.	[47]	Ghana	Relocated coastal residents	Sea-level rise	Anxiety, identity	Cross-sectional	None

HIV, human immunodeficiency virus; PTSD, posttraumatic stress disorder; REFHT, rational emotive family health therapy; RCT, randomized controlled trial.

### Climate events and mental health outcomes

#### Flooding and mental health

Flood-related climate events were the most frequently studied type of environmental exposure. Numerous studies found a psychological impact of climate-related flooding on several population groups. In Ghana, Abass et al. found that exposure to flooding in urban areas significantly increased the risk of psychological distress among both male and female adults (β = 0.030; *p* < 0.005) [22]. A study in Ghana exploring the mental health effects of recurrent flooding in Accra using a Photovoice approach found that residents reported persistent stress and anxiety, particularly among women and children, compounded by inadequate housing and poor access to healthcare services [39]. Furthermore, using the Kessler Psychological Distress Scale (K10), the researchers found that men and younger adults were more likely to experience psychological distress in response to floods. Similar findings were observed among schoolchildren affected by the 2011 northern floods in Namibia. Taukeni et al. found that two years after the flood, over 55% of schoolchildren exhibited significant PTSD symptoms, highlighting the long-term psychological impact of climate-induced flooding [36]. The PTSD symptoms were measured using the Child Trauma Screening Questionnaire (CTSQ), which evaluates re-experiencing and hyper-arousal symptoms, with children scoring 5 and above on a 10-item scale meeting the threshold for clinically significant trauma [29].

Studies assessing the impact of various interventions on flood-induced mental health challenges were limited. A 2023 randomized control trial in Nigeria, assessing the effectiveness of rational emotive family health therapy (REFHT) among cassava farmers, demonstrated a statistically significant reduction in flood-induced symptoms in the intervention group [41]. Another interventional study, also conducted in Nigeria, found that drama and music therapies significantly reduced the symptoms of generalized anxiety disorder (GAD) and PTSD among schoolchildren affected by floods by about 48% [45]. Drama therapy was found to be particularly effective for PTSD.

#### Drought and mental health

In Tanzania, Heaney and Winter’s case study investigated the health perceptions of Maasai migrants and found that drought-induced migration was associated with increased stress, loneliness, and a sense of hopelessness among the migrants [35]. A similar outcome was observed in a qualitative study among Kenyan farmers with HIV who expressed profound emotional distress, including anxiety, sadness, and fear, as a result of crop failure, food insecurity, and disrupted livelihoods following climate-induced droughts [24]. Just like farmers in Kenya, rural farming households in Nigeria reported experiencing depression induced by climate change due to crop losses and their inability to meet basic needs [42].

#### Sea-Level rise and planned relocation

Abu et al. assessed the impact of rising sea levels on the mental health of Ghanaian communities that had been relocated due to coastal erosion [47]. The study found significantly higher anxiety levels and lower well-being among relocated individuals. The same study emphasized that one of the negative impacts of relocation due to rising sea levels includes loss of social cohesion, reduced self-efficacy, and diminished community identity among relocated individuals.

#### Extreme weather events

Two studies in Kenya assessed the mental health impacts of extreme weather events (EWE). Balakrishnan et al. investigated the socio-ecological impact of EWE in two informal settlements in Nairobi, Kenya, and found that participants experienced heightened stress and anxiety due to increased illness, income loss, and disruption to their social networks [46]. There were similar findings among high school students who experienced higher levels of climate-related worry, like anxiety (35%), anger (25%), fear (36%), and powerlessness (22%), and adverse mental health outcomes, including suicidal ideation [43].

### Direct and indirect pathways

#### Direct impacts

Psychological disorders like trauma-related conditions (notably PTSD) were prevalent among children and adolescents exposed to floods in Namibia and Nigeria [36, 45]. Additionally, anxiety and emotional dysregulation were associated with physiological stressors like heat and drought in Kenya and Nigeria [45, 43].

#### Indirect impacts

The indirect impacts of climate change on mental health in the reviewed studies included economic insecurity, disruption of community social cohesion, changes in personal and professional identity, loss of social capital, and fear of future climate-related impacts [20, 24, 42, 47, 38]. Economic insecurity was a key factor, particularly among farmers and rural households, who reported distress due to crop failure and livelihood loss [24, 42]. Beyeler et al. and Abu et al. demonstrated the disruption of community social cohesion, where traditional roles, community support, and trust networks were eroded by climate-related displacements and environmental degradation [24, 47]. Beyeler et al. also qualitatively demonstrated changes in personal and professional identity among men unable to fulfill the expected provider role, leading to diminished self-worth [24]. Finally, the fear of future climate impacts emerged as a theme in two studies, among youth and farming communities, indicating anxiety, helplessness, and eco-paralysis [20, 24].

### Vulnerable populations

#### Children and adolescents

In a survey of 2600 Kenyan high school students, the authors found that emotional reactions to climate change, including anxiety (35%), anger (25%), fear (36%), and powerlessness (22%), were widespread among the students [43]. The same study found a strong association between climate-related worry and suicidality [43]. The study found that students who reported high concern about climate change were significantly more likely to experience suicidal thoughts, plans, and attempts. Similarly, two years after a severe flood in Northern Namibia, over 50% of children aged 12 and below and over 70% of those over 12 reported significant symptoms of PTSD [36].

#### Women and gender considerations

Gender-specific analysis within each study revealed key disparities in the impact of climate change on mental health. A study of European and African French-speaking participants found significantly higher levels of climate anxiety among women participants compared to men across both African and European settings [40]. The trend was particularly pronounced among younger women. A similar outcome was observed in a study of informal settlements in Kenya [46]. The study found that women in those informal settlements experienced high levels of stress and anxiety after experiencing extreme weather events. The psychological burden was exacerbated by environmental burden and socioeconomic marginalization. Also in Kenya, female high school students consistently reported greater emotional distress related to climate change than their male counterparts, particularly in terms of fear and helplessness [43]. A randomized controlled trial in Nigeria indicated that boys showed more significant reductions in PTSD and anxiety symptoms after participating in drama and music therapy for flood-related trauma [45]. This suggests that the effectiveness of mental health interventions may vary by gender.

#### Climate migrants

Two studies highlighted the unique psychological challenges faced by those who migrate due to climate change. The first study of African migrants in Italy found that migrants from African countries with extreme climate vulnerability perceived climate change more acutely and reported a greater loss of social capital [38]. These factors were significantly correlated with symptoms of emotional disorders such as anxiety and depression. The other study of Maasai migrants in Tanzania revealed that migrants experienced elevated levels of stress, loneliness, and unhappiness, attributed to migration-related displacement, role changes, and weakened social networks [35].

#### Rural versus urban populations

Geographic contexts were found to influence the impact of climate-related events on mental health. For instance, Obaniyi et al. observed that rural farming families in Nigeria experienced climate-related depression associated with crop failures and food insecurity, which was exacerbated by limited access to coping resources [42]. There were also rural–urban differences in climate anxiety. Ndetei et al. observed that students in rural areas have significantly higher concern for climate change, with significant implications for mental health [43]. They noted that the difference may be due to higher exposure to environmental degradation and fewer support services in rural settings.

### Persons living with HIV

Chronic health conditions compound vulnerability to climate-induced psychological distress. One study in Kenya explored the experiences of Kenyan farmers living with HIV and found that they faced unique mental health challenges [24]. Climate variability was found to undermine their agricultural livelihoods and exacerbate their financial and emotional stress, thereby threatening their physical and mental well-being.

### Climate anxiety

Climate anxiety is a relatively new term that is gaining more popularity. It refers to the chronic fear and emotional distress associated with the anticipated effects of climate change, even among individuals who have not personally experienced the direct impacts of climate change [26]. This review identified several studies that examine the prevalence, manifestations, and potential mitigating factors of climate anxiety in African populations.

#### Prevalence of climate anxiety across african countries

The studies included in this review highlighted significant and measurable concerns among the general public and specific subgroups across Africa. A multi-country study including Gabon, Rwanda, Morocco, Algeria, and the Democratic Republic of the Congo found that 11.64% of participants reported experiencing climate anxiety frequently [40]. The same study found that over one-fifth of participants reported that climate change anxiety had a significant impact on their daily life activities, such as work and social engagements. Climate anxiety appears to be more prevalent in younger people. Hickman et al.’s global study, which included Nigerian youth, found that 59% of young respondents were either “very” or “extremely” worried about climate change [20]. Specifically, among Nigerian youth, more than 60% expressed high levels of climate-related emotional distress like sadness, helplessness, fear, and powerlessness [20]. The high rate of concern was associated with a high negative perception of government responses and a sense of betrayal, contributing to elevated mental health burden on this demographic.

#### Impacts on healthcare workers

The impact of climate anxiety was observed in healthcare providers. A multicenter study among nursing faculty across Egypt found a negative correlation between climate anxiety and job engagement [44]. The study showed that nurses who reported high levels of climate-related worry also reported lower levels of professional motivation and satisfaction. This suggests that climate anxiety may have occupational repercussions in the healthcare sector.

#### Potential protective factors

One study explored factors that could moderate or reduce the impact or severity of climate anxiety. Notably, Heeran et al. found that among African participants, strong religious beliefs and community-oriented values were associated with reduced likelihood of functional impairment due to climate anxiety [40]. These factors were suggested to reduce emotional distress and foster psychological resilience in the face of climate-related challenges.

## Discussion

Climate change has been shown to adversely affect mental health outcomes [48]. While reviews have examined studies on climate change and mental health, none have focused specifically on African countries, where the impact of climate change is particularly significant [10, 41, 45]. Therefore, this review aimed to evaluate the current literature on climate change and mental health in Africa, identifying trends as well as potential research and policy gaps.

The results of this review indicate that the relationship between mental health and climate change has scarcely been studied in most African countries. We identified only 16 studies that examined this intricate relationship. These findings align with a previous scoping review that found that only five of the 120 studies globally focusing on climate change and mental health were focused on African countries [49]. Most studies were conducted in high-income countries (HICs). While the number of studies has increased since this review was completed, the limited number of studies we found in our review indicates a significant research gap, as we identified studies covering less than 25% of all African countries.

The studies in this review show that, similar to research in HICs, climate change negatively affects mental health both directly and indirectly. Climate change was found to directly affect mental health primarily through extreme weather events such as floods, droughts, and heat waves. Such catastrophic events were found to lead to acute psychological trauma, PTSD, anxiety, and depression. These findings align with previous research that has shown the detrimental effects of climate change on health outcomes globally [17, 51, 50] and suggest a similar mental health trend in Africa.

Climate change was also found to indirectly affect mental health, where climate events led to economic insecurity, disruption of community social cohesion, changes in personal and professional identity, loss of social capital, and fear of future climate-related impacts. These findings align with the conceptual framework proposed by Hayes et al. [52], which outlines direct, indirect, and psychological pathways through which climate change affects mental health [52]. However, our review indicates that in the African context, economic vulnerabilities and social disruptions may play a more significant role as mediating factors compared to HICs, where infrastructure and social safety nets provide greater resilience. Further research is needed to explore this complex relationship.

As the links between mental health and global climate change in Africa become more apparent, evidence-based interventions will be needed to address this challenge in unique African contexts. As has been demonstrated elsewhere, transferring interventions from HICs to LMICs without adequate contextual adaptation may not be successful and sustainable [53]. Only two studies in our review were found to have assessed interventions aimed at improving the mental health of individuals affected by climate change in Africa [41, 45]. This lack of experimental evidence hinders the development of policy responses and health system planning. Given the projected increase in climate-related mental health burdens, there is a pressing need to develop and evaluate psychosocial and community-based interventions that are locally grounded and culturally relevant.

Another major theme of this review is that subgroups such as women, children, migrants, and rural populations are more vulnerable to climate-induced mental health issues. This vulnerability appears to be influenced by several factors, including age-related vulnerability, gender disparities, and geographic and socioeconomic factors. The high prevalence of climate anxiety and PTSD symptoms among African youth reflects findings from global studies, but may be more severe due to limited adaptive capacity in many African contexts [20, 54]. The increased climate anxiety among women across African regions mirrors similar patterns seen globally [26, 55]. This could relate to women’s social roles, limited resource access, and greater household responsibilities during climate crises. The urban–rural divide in climate anxiety, with higher levels in rural areas, contrasts with some findings from HICs where climate concern is often greater in urban centers [56]. This suggests that in many African contexts, direct experience with environmental degradation and agricultural impacts may have a stronger influence on psychological distress than concerns based solely on information. These findings align with the broader climate justice literature, underscoring the intersection between social determinants of health and climate-related impacts [57, 58]. Therefore, interventions and policies should consider the unique climate-induced mental health impacts on these vulnerable populations.

A recent trend in psychological studies on climate is the focus on climate anxiety or eco-anxiety [26, 44, 50]. Eco-anxiety has been described by the American Psychological Association as “a chronic fear of environmental doom,” and it has been linked to a range of symptoms from mild stress to clinical disorders, including anxiety, depression, PTSD, and suicide [50]. Most of the research on climate anxiety has focused on HICs, particularly countries in Europe, North America, and Australia [59]. However, this review identified several studies documenting climate anxiety in LMICs in Africa, indicating that climate anxiety is prevalent in African countries [20, 40, 44]. These studies found that climate anxiety in African countries was particularly prevalent among young people, as has been established in other studies [59–62]. They also suggested that this anxiety can affect daily activities like work and social engagements, as well as lead to lower levels of professional motivation and satisfaction. Therefore, it is crucial to understand and address the detrimental effects of the fear of the future impacts of climate change, as these have direct impacts on the well-being and productivity of the African population, especially the youth. Crucially, further research is needed to understand the extent of climate anxiety across the continent, to develop effective interventions and policies that address its impact.

### Research gaps

While interest in the intersection of climate change and mental health in Africa is growing, the number of studies on this research topic remains limited. Our review reveals critical gaps in geographical coverage, methodological rigor, and thematic focus, as well as a notable lack of research on resilience and adaptation strategies and on post-traumatic growth. In terms of geographical coverage, we did not find a study in most countries. Research on this topic was particularly limited in the Central and North African regions, with most studies concentrated in three countries: Ghana, Kenya, and Nigeria. This research disparity may reflect stronger research infrastructure and international collaboration in these regions.

Another way in which climate change can influence mental health is through its negative impact on sleep [63]. However, no studies in this review explored sleep health. Similarly, there were no studies found on the mediating role of alcohol consumption on the relationship between climate change and negative mental health outcomes.

Furthermore, we found several methodological weaknesses in the existing research. The predominance of cross-sectional designs and the lack of longitudinal studies examining the long-term impacts of climate change on mental health limit the potential for causal inferences. Only two randomized controlled studies examined the effectiveness of mental health interventions. The lack of experimental and interventional studies may limit countries’ abilities to respond effectively to the mental health impacts of climate change. Studies on people with mental health conditions were also lacking.

## Limitations

Several limitations of this review are worth noting. First, we may have missed some studies by including only research conducted in the English language. Some studies may have also been overlooked due to the search strategy employed in the review. Only studies that explicitly linked climate change to mental health or aspects of mental health were included. This inclusion criterion could have excluded literature that highlights associations between extreme weather events, disasters, and other climate-related occurrences and their impact on mental health. Thus, some studies with implied connections between climate change and mental health may have been excluded. Also, the heterogeneity of study designs and measurement approaches makes direct comparison challenging.

We must also acknowledge that the quality of the studies included in this review likely varied, as we did not explicitly assess the quality of the studies themselves. Hence, we cannot comment on whether the current studies should be improved. Nevertheless, the methodology employed effectively accomplishes the overall aim of this study, which is to provide an overview of the current literature and identify gaps in the existing research.

Finally, the limited number of studies identified and included in the review, as well as their geographical distribution, limits the generalizability of this scoping review. Nevertheless, this scoping review highlights crucial insights into the state of climate change and mental health in Africa. Additional research is needed to better understand how climate change is affecting mental health in most countries in Africa. This scoping review could serve as a springboard for such research.

## Conclusion

This review shows that climate change has significant, complex, direct, and indirect effects on mental health across African populations, with certain groups like women, children, migrants, and rural communities being especially vulnerable. The documented rates of both short-term responses and long-term issues highlight the need for more focus on the psychological effects of climate change in Africa. Although limited intervention research poses challenges for developing evidence-based solutions, the identified protective factors offer hopeful directions for creating approaches that fit the local context. Tackling these climate-related mental health issues is a vital part of climate justice and adaptation efforts throughout the continent.

## Patient and Public Involvement

There was no patient or public involvement in this study.

## Additional Files

The additional files for this article can be found as follows:

Supplementary File 1. Boxes.

Supplementary File 2. Preferred Reporting Items for Systematic Reviews and Meta-Analyses Extension for Scoping Reviews (PRISMA-ScR) Checklist.
