# Supplementary material for: Climate Change and Mental Health in Africa: A Scoping Review
Source: Ann Glob Health. 2026 Jan 14;92(1):5. doi: 10.5334/aogh.5110 (PMC12802099; doi:10.5334/aogh.5110)
Supplement: Supplementary File 1. — Boxes. [file agh-92-1-5110-s1.pdf]

Supplemental File 1

**Box 1.** **PubMed search string**
((("Climate Change"[Mesh] OR "climate change"[tiab] OR "global warming"[tiab] OR "extreme weather"[tiab] OR "weather-related"[tiab] OR drought*[tiab] OR flood*[tiab] OR "environmental change"[tiab] OR "climate variability"[tiab] OR "climate disaster"[tiab])) AND ("Mental Health"[Mesh] OR "mental health"[tiab] OR "psychological health"[tiab] OR "mental illness"[tiab] OR "mental disorder"[tiab] OR anxiety[tiab] OR depression[tiab] OR trauma[tiab] OR "psychological distress"[tiab] OR "psychological impact"[tiab] OR "mental wellbeing"[tiab] OR "psychological stress"[tiab] OR "emotional health"[tiab])) AND ("Africa"[Mesh] OR Africa*[tiab] OR "Sub-Saharan Africa"[tiab] OR Algeria OR Angola OR Benin OR Botswana OR "Burkina Faso" OR Burundi OR Cameroon OR "Cape Verde" OR "Central African Republic" OR Chad OR Comoros OR Congo OR "Democratic Republic of Congo" OR Djibouti OR Egypt OR "Equatorial Guinea" OR Eritrea OR Ethiopia OR Gabon OR Gambia OR Ghana OR Guinea OR "Guinea-Bissau" OR "Ivory Coast" OR "Cote d'Ivoire" OR Kenya OR Lesotho OR Liberia OR Libya OR Madagascar OR Malawi OR Mali OR Mauritania OR Mauritius OR Morocco OR Mozambique OR Namibia OR Niger OR Nigeria OR Rwanda OR "Sao Tome and Principe" OR Senegal OR Seychelles OR "Sierra Leone" OR Somalia OR "South Africa" OR "South Sudan" OR Sudan OR Swaziland OR Tanzania OR Togo OR Tunisia OR Uganda OR Zambia OR Zimbabwe)

**Box 2.** **Web of Science search string**
(("Climate Change" OR "climate change" OR "global warming" OR "extreme weather" OR drought* OR flood*) AND ("mental health" OR anxiety OR depression OR trauma OR "psychological distress") AND (Africa OR "Sub-Saharan Africa" OR Algeria OR Angola OR [list of African countries abbreviated]))

**Box 3.** **African Journal Online search string**

("climate change" OR "global warming" OR "extreme weather" OR drought OR floods OR "environmental change") AND ("mental health" OR "psychological health" OR anxiety OR depression OR trauma OR "psychological distress" OR "mental wellbeing")
